# Thermodynamic Characterization of Free and Surface Water of Colloidal Unimolecular Polymer (CUP) Particles Utilizing DSC

**DOI:** 10.3390/polym12061417

**Published:** 2020-06-24

**Authors:** Peng Geng, Ashish Zore, Michael R. Van De Mark

**Affiliations:** Department of Chemistry, Missouri S&T Coatings Institute, Missouri University of Science and Technology, Roll, MO 65409, USA; pgkr4@mst.edu (P.G.); aszbnd@mst.edu (A.Z.)

**Keywords:** Colloidal Unimolecular Polymer (CUP), Differential Scanning Calorimetry (DSC), surface water, density, heat of fusion, thickness, cooling rate, specific heat, melting point depression, counterion condensation

## Abstract

Colloidal Unimolecular Polymer (CUP) particles are spheroidal, 3–9 nm with charged groups on the surface and a hydrophobic core, which offer a larger surface water fraction to improve the analysis of its characteristics. Differential scanning calorimetry (DSC) was performed to determine the characteristics of surface water. These properties include the amount of surface water, the layer thickness, density, specific heat of the surface water above and below the freezing point of water, melting point depression of free water, effect of charge density and particle size. The charge density on the CUP surface was varied as well as the molecular weight which controls the particle diameter. The surface water is proportional to the weight fraction of CUP <20%. Analogous to recrystallization the CUP particles were trapped in the ice when rapidly cooled but slow cooling excluded the CUP, causing inter-molecular counterion condensation and less surface water. The density of surface water was calculated to be 1.023 g/mL to 1.056 g/mL depending on the surface charge density. The thickness of surface water increased with surface charge density. The specific heat of surface water was found to be 3.04 to 3.07 J/g·K at 253.15 K and 3.07 to 3.09 J/g·K at 293.15 K. The average area occupied by carboxylate and ester groups on the CUP surface were determined.

## 1. Introduction

The freezing of water has been an extensively studied thermodynamic property. The freeze thaw of sperm, eggs, cryogenic preservation, crop freezing, food preservation by freezing, paint freeze thaw stability, road and walkway ice and aircraft icing are just a few examples of where ice formation is a critical issue. Surface associated water plays an important role in most of these examples. However, the study of surface water has been difficult due to the low ratio of surface to free water present in a system. Nano particles can offer a significantly enhanced window into surface water due to the high surface water to particle weight ratio.

**Figure 1:**
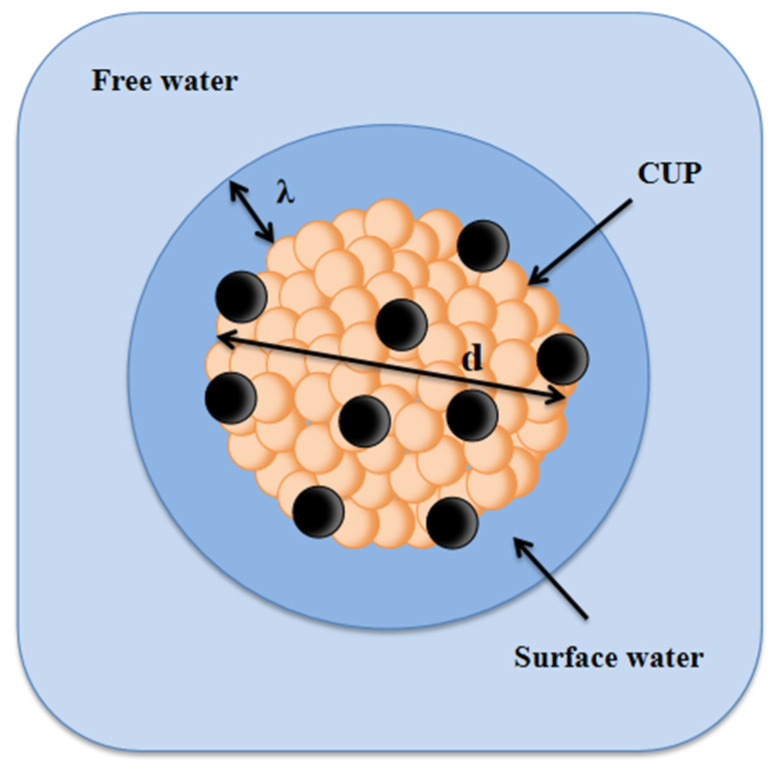
Colloidal Unimolecular Polymer (CUP) particles with surface water.

Colloidal Unimolecular Polymer (CUP) particles are a new class of spheroidal nanoscale polymer particles (3–9 nm) with charged hydrophilic groups on the surface and a hydrophobic backbone [1]. These nano particles can be inexpensively and easily synthesized, particle size can be varied with narrow particle size dispersity by controlling the molecular weight and different surface charge density can be designed with both positive and negative charges, this will provide a very predictable, controllable and reproducible system. Also, CUP particles are spheroidal with charges on the surface but unlike latex particles, CUP solutions are free of surfactant or additives and are zero VOC, making it a promising model for fundamental scientific studies. The CUP surface has a layer of surface associated or bound water, which does not freeze until a much lower temperature than free water [2,3,4,5,6], shown in Figure 1.

**Figure 2:**
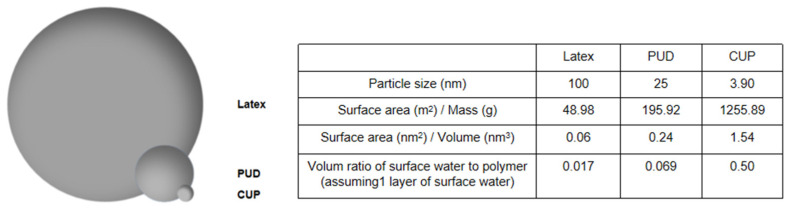
Comparison of latex, polyurethane dispersion (PUD) and CUP (25.4k).

Due to the small particle size, CUP’s surface area per gram is ultra-high, giving a high surface area to volume ratio and surface functionality [7]. The effect of surface water can be neglected when the size of the particle is very large. The size of a typical latex particle is about 100 nm and the diameter of water molecule is only 0.28 nm. Assuming that there is one layer of water bounded on the particle surface, the volume ratio of bound water and latex particle is only 1.69%. However, when the particle is as small as 3.90 nm, the ratio is 49.56% [1], which makes the contribution of surface water much more significant as shown in Figure 2.

These facts make CUP an ideal model for surface water studies, offering a huge advantage over proteins which are limited in size, structure and availability [7]. CUPs can contribute to our understanding of proteins and micelles and also contribute to the study of other materials with water interfaces. Zero VOC CUPs are a very good candidate for future applications including coatings, adhesives, sealants and many others.

The formation of CUP particles is generally accomplished through a water reduction process, according to the Flory-Huggins theory [8]. The process is driven by the polymer-polymer interaction being greater than the polymer-solvent interaction and entropically favored by releasing water analogously to micelle formation. The charged groups repel each other to create the spheroidal shape. Once formed, CUPs are thermodynamically stable in water solution.

**Figure 3:**
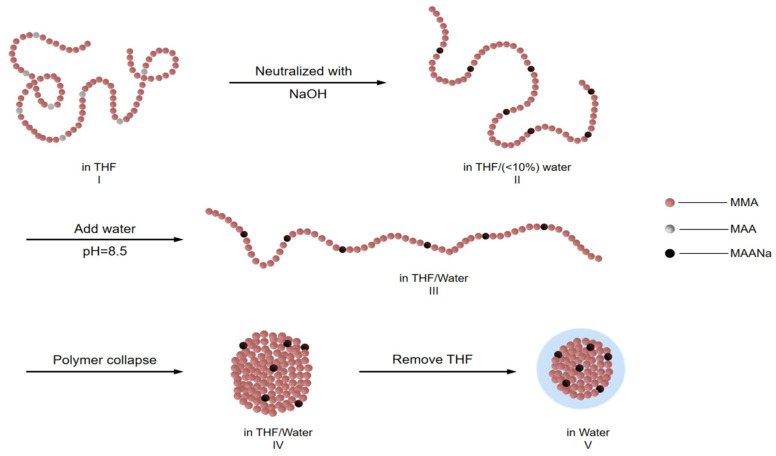
Formation of a typical CUP particle.

CUP solutions are made through a water reduction process, as shown in Figure 3. The brown spheres represent the hydrophobic polymer backbone, each sphere is a methyl methacrylate unit (or hydrophobic monomer). While the gray spheres represent the ionizable carboxylic acid side-chain groups (or methacrylic acid unit). Polymers were dissolved in a low boiling water miscible solvent, like THF, stirred overnight to ensure all polymer chains are in a random coil configuration, Structure I. Base was slowly added to the solution to pH 8.5 forming carboxylate salts and the chains became more extended due to the charge repulsion, Structure II. As pH adjusted water was gradually added, the sodium carboxylate ions became solvated and separated. The repulsion between adjacent ions increased due to the increasing dielectric caused by the added water and the chain extended toward linearity which increased the viscosity [9], Structure III. At a critical water to THF ratio the Mark-Houwink parameter reaches the highest value and the polymer–polymer interaction became greater than the polymer–solvent interaction, the carboxylate groups oriented into the water phase, organizing to produce maximum separation of charge. Hence, water released from the polymer backbone increases the entropy and, the hydrophobic polymer chain collapses into spheroidal shape, Structure IV. Finally, the low boiling solvent, THF was stripped off under reduced pressure. The presence of ionic groups on the surface is the driving force to prevent the particles from aggregating through charge-charge repulsion [10]. Once formed, these colloidal solutions are thermodynamically stable [11]. The ratio of hydrophobic and hydrophilic monomers on the polymer chain (HLB value) is very critical in the unimolecular collapse of the polymer chain during water reduction process. If the chains are too hydrophobic, the collapsed chain tend to aggregate and if the chains are too hydrophilic, the polymer will dissolve or form a more extended or barbell like structure [1,12,13].

The term “surface water” has been used since 1922 [14]. Ever since that, many techniques have been used to investigate the surface water. Previous studies suggest that water molecules were arranged in several layers adjacent to a solid interface changing the properties of the water [15,16,17,18,19,20,21,22,23,24,25]. The state of water has been widely investigated by various techniques.

Van De Mark et al. correlated the surface water with the rheological behavior of CUP particles [10]. They found that from the intrinsic viscosity of the CUP solution, by combining the density and molecular weight of CUPs, the thickness of water layer was estimated to be 0.57 nm, which is similar to the case of protein [2] and cylindrical nanopores [26].

Nuclear magnetic resonance (NMR) is another method which has been used to examine surface water. The mobility of surface water molecules is lower than free water molecules, therefore NMR probing of the mobility has been used to verify the existence of structured water around colloidal polyvinyl acetate (PVA) with particle size of 0.13 and 0.8 micron used by Clifford et al. By measuring the spin lattice relaxation time constants of protons, they concluded that the total amount of bound water per unit surface area was larger for larger particles [27,28,29].

Katayama et al. investigated the states of water in a polyacrylamide gel using spin-lattice relaxation time constant measurements and concluded that the macromolecule must have a hydrophilic substituent to be able to capture surface water and the amount of surface water remarkably depends on the amount and the nature of the substituents [30]. Van De Mark et al. used the same method to determine the surface water thickness of CUP particles, which ranged from 0.19 nm to 0.693 nm and it was found to be both molecular weight and temperature dependent [31].

Quasielastic neutron scattering (QENS) has been used for both organic and inorganic water-containing materials. Mamnontov used neutron scattering to investigate the vibrational dynamics of surface water in ZrO_2_. The rotational diffusion of surface water molecules was found to be slower by about a factor of 2 when compared with free water and the residence time for translational diffusion of surface water was about 40 times longer than free water. It was proposed that there were about two hydration layers on top of the layer of surface OH groups [32].

Toney et al. measured the water density profile perpendicular to silver (III) at two voltages by using X-ray scattering and found that the first inner layer of surface water has a greater density compared with free water, 1.1 (−0.23 V) to 1.8 (+0.52 V) water molecules per Ag atom for surface water, while they expected 0.8 water molecules per Ag atom, based on the density of free water. Which indicated that the hydrogen-boding network changed and resulted in very different properties in this layer from those in free water [33,34]. 

Drost-Hansen et al. studied the water adjacent to silica surfaces by using differential thermal analysis (DTA). They observed thermal property changes in the slope and displacements of the baseline in thermograms and small endothermic peaks in heating curves. These peaks were associated with structural phenomena changes of the water at silica/water interface, indicating the properties of water close to solid interfaces are very different from free water [35].

Velazquez et al. described the interaction of water with hydrophilic materials indicating that a portion of water was firmly bounded to individual sites. By using infrared spectroscopy, the bound water amount on a methylcellulose film was identified. It was reported that the bending vibration mode of bound water shifted toward lower frequency by about 15.5 cm^−1^. When the humidity increased, the bound water content remained at below 5 % [36].

Differential scanning calorimetry (DSC) has been widely used to investigate detailed information on the state of water by distinguishing the amount of freezable water from non-freezable water for many water absorbed system [37,38,39,40,41,42,43], Ross [44], Bushuk et al. [45] and Biswas et al. [46] all concluded that the amount of unfrozen water per gram of solute increased with the increasing dilution, this dependence was more obvious for concentrations smaller than 1 M. 

Berlin et al. studied whey protein systems by using DSC and determined that 0.5 g of water per gram of whey protein would not freeze at −40 ℃. By adding lactose and salts, the unfreezable water increased as the concentration of lactose and salts were increased, which varied between 0.5 and 1.2 g of water per gram of solids [47,48]. Hatakeyema et al. quantitatively calculated the amount of bound water in poly(vinyl alcohol) hydrogel using DSC. It was reported that each hydroxyl group of PVA can associate with 1–1.5 molecules of non–freezable water and 5–6 molecules of freezable bound water [49]. Ostrowska-Czubenko et al. analyzed the state of water in chitosan hydrogel membranes and found that freezable water content increased linearly with the water uptake, while non-freezable water content remained constant beyond critical water content value. The non-freezable surface water ranged from 0.47 to 0.65 g per gram of dry membrane [50]. Muffett et al. investigated the amount of unfrozen water in soy proteins by DSC and reported that the unfrozen water amount increased in the range of 0.09–0.14 g of unfrozen water per gram of total water, with the increasing of total water [51]. Garti et al. determined the thickness of bound water layer in the n-dodecane/1-pentanol/C_12_(EO)_8_ system to be 0.5 nm by using DSC [40]. Kobayashi et al. determined the thickness of fully hydrated dimyristoylphosphatidylcholine to be 3.2–3.4 nm, using DSC, X-ray and densimetry [52].

Many of the previous surface water studies utilizing DSC gives confidence to investigate surface water properties of CUP system. DSC has many advantages over other techniques for evaluating the surface water behavior. First, DSC is the most common thermal analysis technique to obtain heat content change (enthalpy) and heat capacity with ease and speed. Second, DSC can hold a precise temperature with no drift and it can be used for kinetic studies in a faster and more straightforward way than other methods [53,54,55]. In this study, using hermetically sealed pans and sample size of 30 mg, accurate water melting and specific heat data should be readily obtainable for even low weight fraction of CUP solutions.

CUP opens a window to investigate the thermodynamics of surface water and free water for particulate systems. This work presents a primary study of the CUP surface water properties utilizing DSC. It includes—synthesis and production of CUP through water reduction; determination of the thickness of surface water of CUP by measuring the heat of fusion and particle size; determine the actual density of surface water; determine the specific heat of surface water; evaluate the effect of cooling rate on the amount of surface water; determine the average surface area of functional groups on the CUP surface by knowing the melting point depression of CUP solutions.; study the relationship between CUP surface water and molecular weight and charge density in ions per nm^2^. This work represents the first comprehensive study of the functionality and size effects on CUP surface water behavior during freeze/thaw conditions.

## 2. Materials and Methods

### 2.1. Materials

Methyl methacrylate (MMA), methacrylic acid (MAA), 2,2′-azobis(2-methylpropionitrile) (AIBN) and 1-dodecanethiol were purchased from Aldrich (St Louis, MO, USA). MMA was purified by washing with a 10% (w/w) solution of sodium bicarbonate, followed by rinsing with de-ionized water and then brine and then dried over sodium sulfate and filtered. Copper (I) bromide was added to the MMA as an inhibitor and simple distillation under nitrogen was carried out. MAA was purified by distillation with copper (I) bromide under reduced pressure. THF was dried and distilled under protection of nitrogen. AIBN was re-crystallized from methanol and 1-dodecanethiol was used as received.

### 2.2. Synthesis of Poly(MMA/MAA) Copolymer

Polymers were synthesized by a free radical polymerization method in tetrahydrofuran (THF) [21]. To a 2 L three neck flask, 10.40 mol of THF was added. Methyl methacrylate (MMA) and methacrylic acid (MAA) were used with various molar ratios. 1-Dodecanethiol was added as a chain transfer agent based on desired molecular weight of polymer. The initiator AIBN was then added, 0.0007 times the total moles of monomers. The reaction was carried out under nitrogen, with condenser and a gas outlet adapter connected to an oil bubbler to allow a positive flow of nitrogen throughout the polymerization. The mixture was heated slowly to reflux under stirring for 24 h. The polymer solutions were then cooled to room temperature and part of the THF was removed by rotovap. Finally, the polymer was precipitated in cold deionized water under high shearing rate and dried in a 323.15 K oven under vacuum for 24 h. Table 1 shows the component amount for the synthesis of Polymers 1–7.

### 2.3. Water Reduction Method

To a 500 mL Erlenmeyer flask were added 10 g of dry polymer, 40 g of THF and stirred for 12 h to let the polymer chains fully obtain a random coil configuration. Based on the measured acid number, 1 M NaOH solution was slowly added to neutralize the solution to pH 8.5 by peristaltic pump at the rate of 1.24 g/min. Then 90 g of deionized water modified to pH = 8.5 using 1 M NaOH solution was then gradually added by peristaltic pump at the rate of 1.24 g/min. The pH of system was maintained at 8.5 throughout the process of water reduction. Majority of THF was stripped off by rotovap and the sample was left in vacuum to remove the last trace of THF, giving zero VOC CUP solution. The clear solutions were then filtered through a 0.45 micron filter to remove any extraneous trace particulate contaminants, the typical loss on filtering was less than 0.05% of the solids by weight. The CUP weight percent solids was determined and the CUP solution was either diluted by adding pH modified water or concentrated under vacuum to prepare the different CUP solids fractions needed for the study.

## 3. Characterization of Polymers

### 3.1. Absolute Molecular Weight of Copolymers

Gel permeation chromatography utilized a Viscotek model 305 manufactured by Malvern Corp. system (Westborough, MA, USA). The GPC was equipped with a triple detector array TDA305—refractive index detector, low and right-angle light scattering detector and intrinsic viscosity detector, the column, two PAM-505 from PolyAnalytik (London, Ontario, Canada) with a guard column, the column size is 7.5 mm (ID) ×300 mm (L). Polymer samples were prepared to 2 mg/cc. Injection volume was 100 μL and THF flow rate was 0.5 ml/min.

### 3.2. Density of CUP Solution

Densities of CUP solutions were directly measured by density meter (DDM 2911 plus by Rudolph Research Analytical (Hackettstown, NJ, USA), at various weight fractions at 298.15 K. The accuracy is 0.00001 g/cm^3^. 

### 3.3. Density of Dry CUP Polymer

The solutions of CUP were dried in a vacuum oven, heated at 323.15 K with the presence of solid sodium hydroxide to absorb carbon dioxide. After the clear crystal-like material formed, the sample was then heated to 383.15 K to constant weight. The densities of the dry CUPs were measured by a gas displacement pycnometer, Micrometritics AccuPycII 1340. The temperature was controlled at 299.04 ± 0.04 K. Twenty five readings were made for each sample and the results were reported by average and standard deviation.

### 3.4. Acid Number (AN)

The acid number of the copolymers were determined by the titration method ASTM D974 and reported in mg of KOH/g of polymer sample. The method was modified by using potassium hydrogen phthalate (KHP) in place of hydrochloric acid and phenolphthalein in place of methyl orange. THF was used as the solvent of the titration.

### 3.5. Viscosity of CUP Solution

Viscosity of CUP solution was measured based on ASTM D445, ASTM-D446 and ISO 3104, 3105. Ubbelohde capillary viscometer J-340 from Cannon instrument company was used to determine the viscosity of CUP solutions at two different temperatures—298.15 K and 302.15 K. Before each measurement, CUP solution was transferred to the Ubbelohde capillary viscometer and kept in a constant temperature water bath at 298.15 ± 0.1 K for 20 min with plastic wrap covering on top of the viscometer to prevent potential evaporation and carbon dioxide contamination of the solution. A stop watch with 0.01 s precision was used to monitor the elution time and each measurement was repeated for at least three times and the error being less than 0.5%. Absolute viscosity was then calculated by Equation (1).

(1) η=t⋅ρ⋅c,

where η is the viscosity of CUP solution (cP), t is the elution time (s), ρ is the density of CUP solution (g/mL) and c is the Ubbelohde capillary viscometer constant (0.009749 mm^2^/s).

### 3.6. Particle Size of CUP

Particle size was measured by dynamic light scattering, using Microtrac Nanotrac 250 particle size analyzer from Microtrac with a laser diode of 780 nm wavelength and 180° measuring angle. The viscosity of solution was used, due to high weight fraction of CUP having high viscosity that caused by electronic repulsion [10] and low weight fraction of CUP produce very poor light scattering efficiency and a weak signal, because of small particle size, around 5 nm. The CUP solutions were diluted to 10% weight fraction by pH adjusted Mili-Q ultrapure water with resistance of 18.3 MΩ. The particle size was calculated by Stokes-Einstein Equation (2).

(2) $$D=\frac{k_{B}\cdot T}{6\pi \cdot \eta \cdot r},$$

where k_B_ is the Boltzman constant, T is the absolute temperature of solution, η is the viscosity of solution and r is the radius of the CUP particle. 

### 3.7. Differential Scanning Calorimetry

Differential scanning calorimeter from TA instruments Q2000 was used to measure the heat of fusion, specific heat and melting point depression of CUP solutions. About 30 mg of CUP samples were sealed in the Tzero Hermetic pan from DSC Consumables Inc., then cooled to the target temperature at a series of cooling rates, isothermal for 10 min and heated up to 313.15 K at 3 K/min rate. The mass of sealed pan was measured before and after each measurement with the result being considered valid if the mass difference was smaller than 0.001 mg. 

## 4. Results and Discussion

### 4.1. Polymer Synthesis and Characterization

It was found that polymers with molecular weight lower than 13,000 g/mol tended to be unstable, due to insufficient electrostatic stabilization causing the polymer to aggregate. Polymers with very high molecular weight, higher than 200,000 g/mol might have issue during water reduction process due to chain–chain entanglement. At high molecular weight the reduction solution must be very dilute since as the molecular weight increases the chance for chain entanglement increases therefore the solutions must be diluted. Thus, seven polymers with different molecular weights (22,700–122,500 g/mol) and different monomer ratios were synthesized and successfully made into CUP solutions. Polymer 1, 2 and 3 have different molecular weight but the same monomer ratio, 9 mol MMA to 1 mol MAA. In order to investigate the behavior at very low charge density values, Polymer 6 and 7 were synthesized with a large monomer ratio. To understand the effect of the molecular weight, monomer ratio and ions per nm^2^ (surface charge density) effect on the surface water properties, Polymer 4 and 5 were designed to have different molecular weight but same charge density in ions per nm^2^ as Polymer 2. The charge density ρ_v_ was determined by Equation (3).

(3) $$\rho_{v}=\frac{M_{W}}{4\pi \cdot r^{2}(n_{1}\cdot M_{MMA}+n_{2}\cdot M_{MAA})},$$

where ρ_v_ is the charge density in ions per nm^2^, M_w_ is the molecular weight of CUP polymer, r is the radius of the CUP particle, n_1_ is the moles of MMA and n_2_ is the moles of MAA used per average repeat unit, M_MAA_ is the molecular weight of monomer methacrylic acid, M_MMA_ is the molecular weight of monomer methyl methacrylate. Polymer 6 and Polymer 7 were synthesized to have a much higher monomer ratio which was able to investigate the behavior at very low charge density values. 

The molecular weight, acid number, particle size and density of the polymers are listed in Table 2. The densities of the dry polymer increased with the increasing of molecular weight, because when molecular weight is larger, the number of polymer chain end groups decrease, result in less free volume and higher density [56]. All polymers had consistent acid numbers with respect to the monomer feed.

The polymers were then reduced, the THF removed and the CUP solutions were concentrated. The samples were then measured for particle size and validated as to single chain particles due to a match in particle size with the calculated size and distribution from the absolute molecular weight [1]. The samples were either concentrated or diluted into different weight fractions for DSC analysis.

### 4.2. Effect of Cooling Rate and Ice Formation

The DSC measurements were done using about 30 mg of CUP solution in Tzero hermetically sealed pans. Enough head space was allowed to avoid rupture or leaking during the freeze thaw cycle and large enough to give an accurate measurement. DSC samples were cooled to 233.15 K and held for 10 min to ensure all freezable water froze and then heated to 313.15 K to allow all the ice to melt without causing leakage. It was difficult to precisely obtain accurate area of the ice formation peak due to some super-cooling during the temperature lowering scan. Therefore, the data presented here represents the heating, melting process only. The mass of the DSC pan was measured before and after to ensure that no water had been lost during the cycle, only runs with losses of less than 0. 001 mg were used.

**Figure 4:**
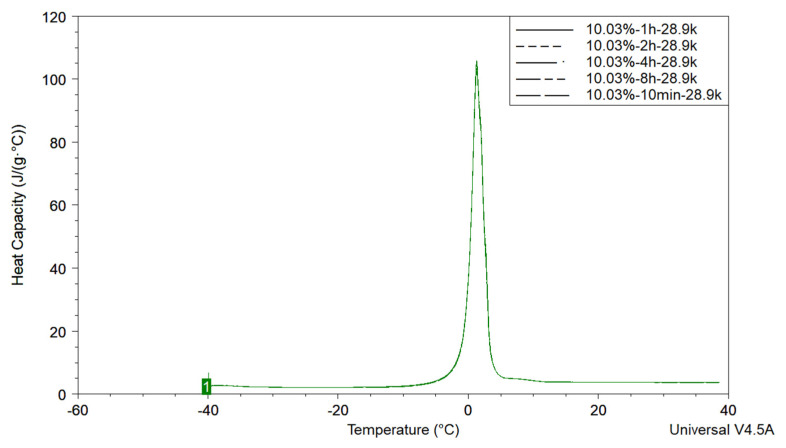
Heat of fusion of 10.03% Polymer 1 with different isothermal time.

To evaluate the time required to freeze the sample as well as to determine if the surface water will slowly leave the outer surface and freeze, samples of a 10.03% 28.9k CUP solution, Polymer 1, were cooled to 233.15 K at 10 K/min, isothermal for 10 min, then heated up to 313.15 K at 3 K/min. The same sample was also measured following the same protocol, except the isothermal time was changed to 1 h, 2 h, 4 h and 8 h. The endothermic peaks represented the measured heat of fusion of freezable water. The same area and shape indicated that all freezable water was able to completely freeze within the 10 min isothermal period, shown in Figure 4. Thus, 10 min was chosen as the experimental isothermal time. 

**Figure 5:**
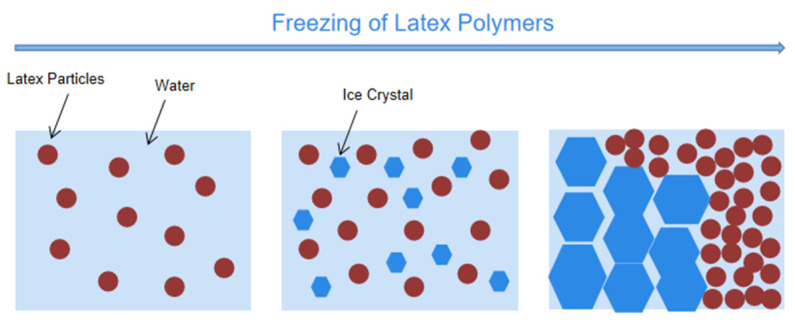
Freezing of Latex polymers.

Ice forms typically near 273 K, however, the endothermic peak is somewhat broad so is the freezing peak making baseline determinations near 273 K ± 10 K difficult. Evaluation of the lowest temperature needed was evaluated by running scans at with 10.40% 28.9k CUP solution cooled to 233.15 K, 243.15 K and 253.15 K at 10 K/min from room temperature, isothermal at each temperature for 10 min. Then the sample was heated up to 313.15 K at the rate of 3 K/min. The heat of fusion was unchanged over this range. Which implied that the 233.15 K isothermal temperature could assure all freezable water froze. When water-based latex resins are exposed to cold temperature conditions, water will start to freeze and form ice crystals excluding the latex. As the ice crystals grow, there is less and less liquid water existing in the solution and therefore the weight fraction of latex particles increases in the remaining liquid and the particles are forced close together [57], shown in Figure 5.

Thus, in theory, CUP solutions should perform in a similar way. If the CUP solution was cooled at an infinity slow rate, the distance between each CUP particles will become a minimum, reaching maximum inter-molecular counterion condensation effect and result in the minimum amount of surface water, regardless of initial weight fraction of CUP particles. 

In the literature, different freezing methods result in different supercooling effects [58]. For normal protein freeze-drying process, the fastest cooling rate can be obtained by liquid nitrogen freezing with a small volume of sample, that gives the most supercooling; while the lowest supercooling is for the pre-cooled shelf method [59,60,61]. 

In order to understand the effect of the cooling rate on the measurement. Variation in the cooling rate was evaluated analogously for Polymer 1. The DSC of CUP solutions were cooled at 0.1 K/min, 0.2 K/min, 0.5 K/min, 1 K/min, 2 K/min, 3 K/min, 5 K/min, 7 K/min and 10 K/min. Eight weight fractions of Polymer 1 CUP solutions were cooled to 233.15 K at different rates, then isothermal for 10 min and heated to 313.15 K at the rate of 3 K/min. The amount of non-freezable surface water per CUP particle was calculated by Equation (4).

(4) $$m_{SW}=\frac{(\frac{\Delta H_{W}-\Delta H_{FW}}{\Delta H_{W}}-X_{CUP})\cdot M}{N_{A}\cdot X_{CUP}},$$

where m_sw_ is the mass of surface water per CUP particle in grams, N_A_ is Avogadro constant, ΔH_W_ is the heat of fusion of water (333.5 J/g), ΔH_FW_ is the heat of fusion of freezable water in CUP solution, M is the molecular weight of CUP polymer, X_CUP_ is the weight fraction of CUP in the solution.

**Figure 6:**
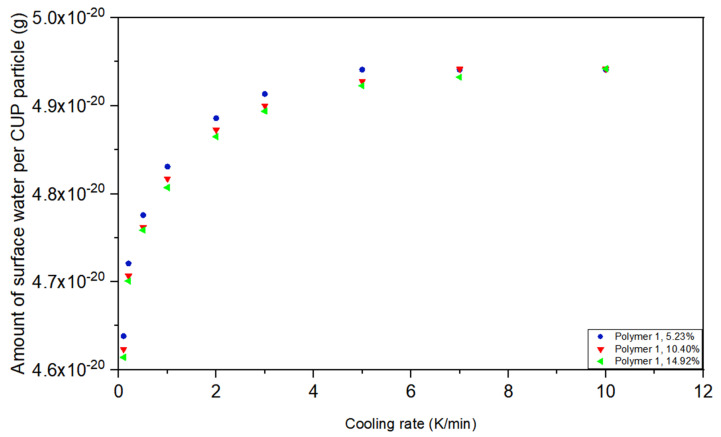
Amount of surface water per CUP particle of Polymer 1 at 5.23%,10.40% and 14.92% at different cooling rates.

In order to demonstrate the effect of cooling rate on the amount of surface water, the DSC measurement of Polymer 1 solutions was conducted at 10 K/min cooling rate. Results showed that the amount of surface water was constant at concentrations below where Manning condensation occurs, Figure 6. However, as the cooling rate was reduced the amount of surface water measured decreased and showed a small concentration dependency. As the concentration of CUP increased the surface water decreased only slightly. During the freezing process, ice nucleates and the crystals grow. In a supercooling situation, when ice nucleation occurs growth is extremely rapid and traps the CUP particles in the crystal matrix before significant diffusion can occur. However, when slow cooled the ice growth was slower and allowed time for CUP diffusion and subsequent Manning condensation to occur [62]. When this charge condensation occurs the amount of surface water drops. At 0.1 degrees per minute and 5.23% CUP, the amount of surface water is 4.64×10^−20^ g per particle but at 10 degrees per minute it was 4.94×10^−20^ g per CUP particle, the drop in surface water was only 6.07%. All the CUPs in this study were polymerized from methyl methacrylate and methacrylic acid, the surface charged groups originated from neutralization of carboxylic acid by NaOH. The effective charge on the surface is subject to the dissociation equilibrium and there are two counterion condensations that exist in this system, inter-molecular counterion condensation and intra-molecular counterion condensation [63,64,65]. Inter-molecular counterion condensation occurs between particles when the weight fraction of CUP particles is high and intra-molecular counterion condensation occurs where the charge density of a single particle is high. Slow cooling rates allowed more time for CUP particles to migrate, when CUP particles were forced to become closer, the inter-molecular counterion condensation effect increased which reduced the number of effective charges on CUP’s surface, releasing part of the surface water where it became freezable. The counterion condensation effect was greater for higher concentrations of CUPs. This small difference is likely caused by the increased chance of charge condensation in the more crowded higher concentration.

**Figure 7:**
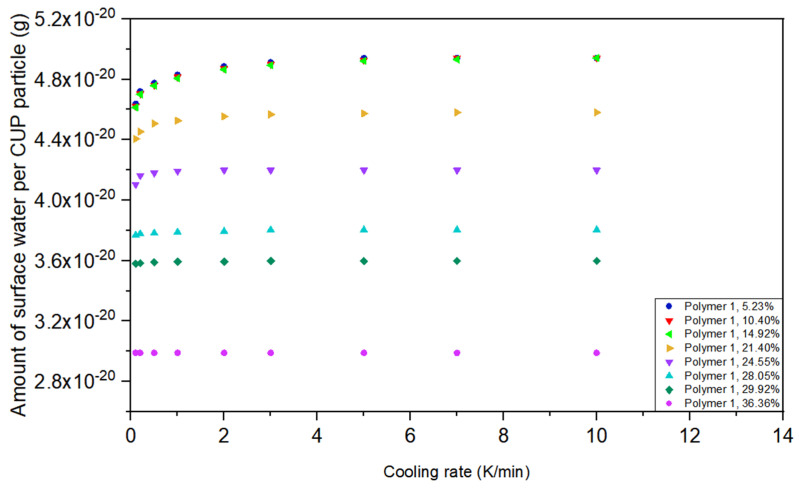
Amount of surface water per CUP particle of Polymer 1 at different weight fraction at different cooling rates.

Solutions with 21.40% and higher weight fraction CUP showed less surface water per particle at 10 K/min, Figure 7. The cause for this decrease was inter-molecular counterion condensation. When the weight fraction of CUP is high, with short distances between CUP particles, inter-molecular counterion condensation existed even before the cooling process. The inter-molecular counterion condensation also reduced the ice formation driven charge condensation which occurs during slower freezing rates. As the concentration increases the effect of slow freezing is reduced and at about 36% the surface water is a constant being independent of the cooling rate. It is important to note that the viscosity of the CUP solution is concentration dependent and rises rapidly due to the onset of Manning condensation [8,66]. The ion-ion repulsion reduces the diffusion rate of the CUP particle and thus makes it harder to move away from the advancing crystals of ice being formed and the CUP becomes an inclusion.

**Figure 8:**
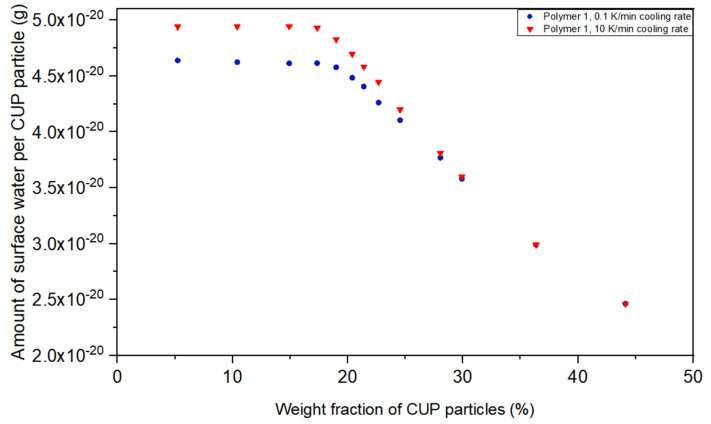
Amount of surface water per CUP particle of Polymer 1 at different weight fraction at 0.1 K/min and 10 K/min cooling rate.

Direct comparison of the amount of surface water of the solutions with different CUP particle weight fractions are shown in Figure 8. The effect of the cooling rate decreased with the increasing of CUP particle weight fraction and gradually disappeared. The initial concentration independent region was again below the inter-molecular counterion condensation region. At approximately 20% the contribution due to the inter-molecular counterion condensation and subsequent viscosity effect obviously controlled the system. When the gel point is approached the particles cannot significantly move and the surface water reaches a point where the rate of cooling does not affect the result. Figure 8 illustrates the effect of 0.1 K/min versus 10 K/min cooling rates. At low concentration the surface water per cup was a constant until Manning condensation occurs then as the concentration increases the differences reduce to zero at the gel point.

The packing of spheroidal materials is most likely to form either random close packing or hexagonal close packing. The maximum volume fraction 0.634 is for random close packing and for hexagonal close packing it is 0.7405 [67,68,69,70,71]. CUP solution is able to approach hexagonal closing packing with the volume fraction of CUP particle being 0.7405 including surface water. Due to the small particle size and relatively low density the Brownian motion of CUP can help to move the particles to a position where the electrostatic repulsion and Brownian motion reach a balanced stable structure. Based on the previous publication by Van De Mark et al. [8], the weight fraction of CUP particle for each packing model can be calculated by Equations (5)–(7).

(5) $$\varphi =\frac{\rho_{s}\cdot X_{CUP}}{\rho_{p}}$$

(6) $$\varphi_{\max}{\left(1+\lambda /r\right)}^{3}=0.634$$

(7) $$\varphi_{\max}{\left(1+\lambda /r\right)}^{3}=0.7405,$$

where ɸ is the CUP volume fraction, ρ_s_ is the density of CUP solution, ρ_p_ is density of CUP particle, X_CUP_ is the weight fraction of CUP, ɸ_max_ is maximum volume fraction, λ is the thickness of surface water, r is radius of the CUP particle.

For CUP solutions of Polymer 1, the weight fraction of CUP particles was 36.16% when reaching random closing packing, which is the gel point and 43% for hexagonal close packing. When the weight fraction of CUP particles was close to 30%, the cooling rate effect on the amount of surface water was almost gone, due to the very high viscosity and close to maximum inter-molecular counterion condensation. The surface water difference between the two cooling rates approached zero when the weight fraction of CUP particles is close to 43%, at this point and the particle is very difficult to move and any free water will be slow to migrate. It is also the point where CUP particles reaches maximum inter-molecular counterion condensation over the concentration range of 20% to 43%, CUPs the surface water amount drops by 47.55% at 10 K/min and 45.07% at 0.1 K/min.

The behavior of CUP solutions were similar to that of latex resins in that as the ice forms the particles are forced together. The higher charge density on the surface and the increased surface area create an increased amount of surface water which does not freeze. The same effects should be observed in globular proteins and other nanoscale particles in water as well as in vitro and vivo materials when frozen.

### 4.3. Weight Fraction of Surface Water

In order to minimize the effect of counterion condensation in the remaining portion of this work, all the CUP solutions were cooled at 10 K/min, which is the highest cooling rate the DSC was capable of. The isothermal time was kept at 10 min and heated to 313.15 K at the rate of 3 K/min. Furthermore, since the CUP samples were neutralized by NaOH solution, heat of fusion of NaOH modified water sample was measured. Since there was no difference in the heat of fusion and melting point depression between NaOH modified water and deionized water (333.5 J/g), the deionized water was used as the standard. Different molecular weight polymers were made into CUP solutions and were prepared to various weight fractions. The heat of fusion of each solution was measured by DSC.

**Figure 9:**
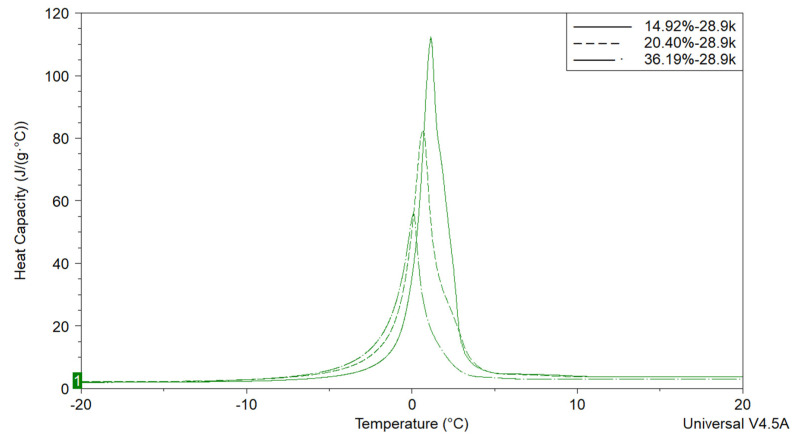
Heat of fusion of Polymer 1 solution at various weight fraction.

Figure 9 shows the DSC endothermic peak of Polymer 1 CUP solutions with three weight fractions. It was obvious that with the increasing of the weight fraction, the endothermic peaks shift to lower temperature, this can be explained by Raoult’s law [72]. CUP solutions contain only CUP particles, free water and surface water. The surface water does not freeze until a very low temperature and the free water which freezes like normal water. The endothermic peak for all the samples exhibited only a single peak. Thus, the observed difference in heat of fusion, between a water sample and a CUP solution, is due to the CUP particles and a fraction of water that does not freeze in the DSC measurement [46]. Therefore it is possible to determine the weight fraction of surface water utilizing the heat of fusion by DSC. For CUP samples, the weight fraction of free water can be determined by dividing the heat of fusion of the CUP solution by that of deionized water, which was used as a standard, Equation (8).

(8) $$X_{FW}=\frac{\Delta H_{FW}}{\Delta H_{W}},$$

where X_FW_ is the weight fraction of freezable water, ΔH_FW_ is the heat of fusion of freezable water obtained from DSC, ΔH_W_ is the heat of fusion of water, 333.5 J/g. The surface water weight fraction, X_SW_ can be calculated knowing the weight fraction of CUP, X_CUP_ and X_FW_, Equation (9).

(9) $$X_{SW}=1-X_{FW}-X_{CUP}.$$

Combining Equations (8) and (9) to obtain Equation (10).

(10) $$X_{SW}=1-X_{CUP}-(\frac{\Delta H_{FW}}{\Delta H_{W}}).$$

**Figure 10:**
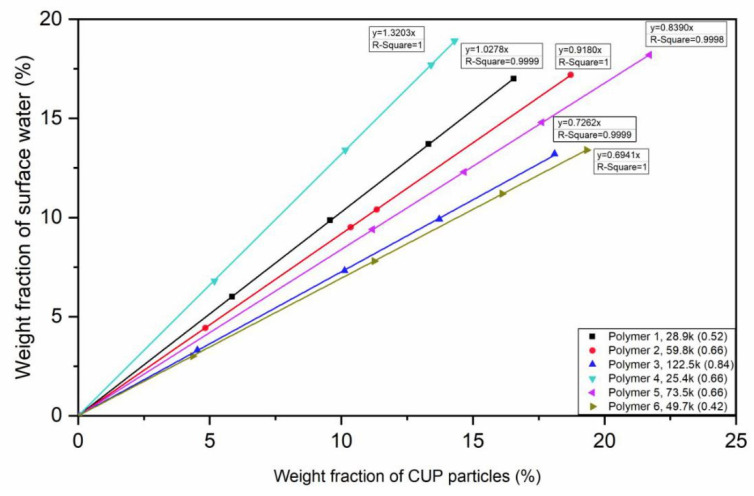
Weight fraction of surface water of different CUPs with different monomer ratio vs weight fraction of each CUP. Note: The box legend is Polymer, molecular weight and charge density.

From Equations (8)–(10), the weight fraction of surface water of each CUP solution was determined. Figure 10 shows the weight fraction of surface water vs weight fraction of the CUP particles.

The results indicate that there was a significant amount of non-freezable water in this system. The amount of water was linearly dependent upon the weight fraction of CUP over the range shown. Thus, the amount of water was constant per particle. Comparing Polymer 1, 2 and 3, that have a 9:1 MMA:MAA ratio, the higher molecular weight result in a lower slope, due in part to the lower surface area per gram of polymer. However, there is another variable, the charge density of ions on the CUP surface. As the molecular weight increases at a constant MMA:MAA ratio the surface charge density increases since all the charged groups will attempt to be on the surface. It would be expected that more charge density the more surface water should be observed. 

Molecular weight of Polymer 4 is slightly larger than Polymer 1, the weight fraction of surface water should have been similar if the surface water is only dependent upon surface area and not on charge density. However, the charge density of Polymer 4 is much higher than polymer 1 and the surface water was significantly higher. This increase indicates that the surface water is dependent upon charge density. In addition, by comparing Polymer 2 and 5, with similar charge density, Polymer 2 has smaller molecular weight, so that it has more surface area and also more surface water. The conclusion can be made that both molecular weight and charge density play important roles in affecting the weight fraction of surface water. As for the same monomer ratio, larger molecular weight CUP particles will have higher charge density and if the monomer ratio is different, with similar molecular weight, the surface water weight fraction is proportional to the ions per nm^2^ and higher charge density gives a higher surface water weight fraction. When the charge density is the same, higher molecular weight CUP has less surface water weight fraction that is, the surface area defines the surface water weight fraction. 

**Figure 11:**
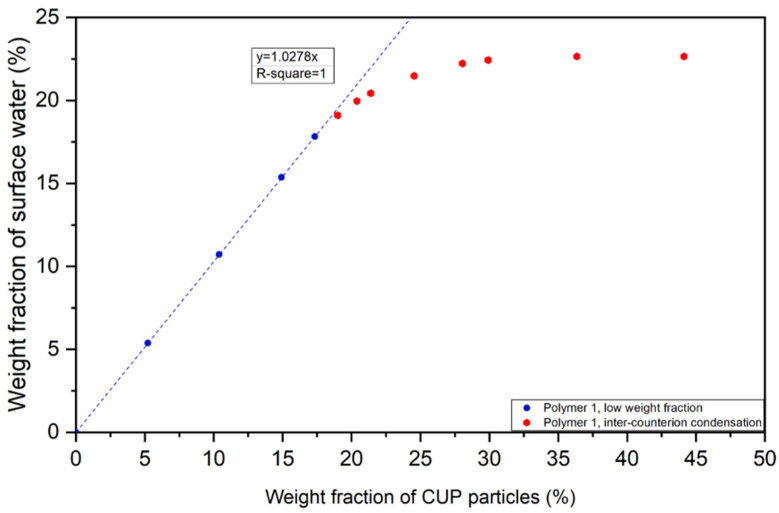
Weight fraction of surface water vs CUP particles of Polymer 1.

All the CUP solutions discussed in Figure 10 are below the concentration where Manning condensation occurs. Polymer 1 was used to evaluate higher concentrations as is shown in Figure 11. At concentrations above 20% the weight fraction of water begins to plateau at about 35%. Increasing the concentration causes an increase in the inter-molecular counterion interaction as the distance between ions becomes shorter. This interaction causes Manning condensation and reduces the effective charge on the surface. With less charge, the hydration layer decreases. Looking at Figure 10 and Figure 11, the linear behavior at low concentration can be used to define when Manning condensation becomes significant by noting where the plot becomes non-linear.

### 4.4. Surface Water Density

Surface water has been found to have a greater density than bulk or free water [10,33,34]. To gain a deeper understanding of CUP surface water, the density of the CUP solutions were measured by high precision density meter at various weight fractions of CUP particles. Values of 1/ρ_s_ were plotted against weight fraction of CUP particles. The reciprocal of density of CUP solution was found to have a linear relationship with the weight fraction of CUP particles at low concentration [8].

**Figure 12:**
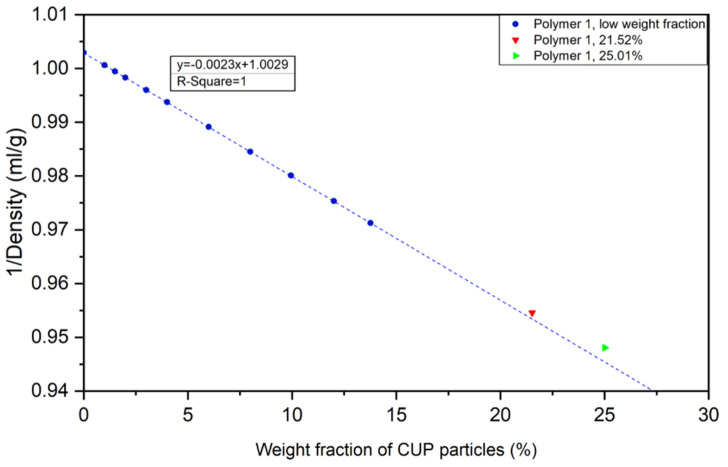
Dependence of 1/ρ_s_ on weight fraction of Polymer 1 CUP solution.

Figure 12 shows polymer 1 as an example. It was observed that with the increasing of weight fraction of CUP particles, the reciprocal of density had an excellent linear regression fit up to about 20%. However, at higher concentrations the data deviated from linearity. This deviation can be explained by the increased weight fraction of CUP particles shortening the distance between the charged particles causing inter-molecular counterion condensation. The condensation reduces the amount of bound water per particle and thus alters the observed density. Higher concentrations could not be accurately measured for density due to their high viscosity.

In a given CUP solution, there are three components—free water, surface water and CUP particles. The combination of volume of CUP solid, volume of surface water and volume of free water is equal to the volume of the solution. By knowing the weight fraction of surface water, free water and CUP solids, as well as the density of CUP solid, free water and solution, the density of surface water can be determined by Equation (11).

(11) $$\frac{1}{\rho_{S}}=\frac{X_{FW}}{\rho_{FW}}+\frac{X_{SW}}{\rho_{SW}}+\frac{X_{CUP}}{\rho_{CUP}},$$

where ρ_s_ is the density of CUP solution, ρ_sw_ is the density of surface water, ρ_FW_ is the density of free water, ρ_CUP_ is the density of CUP particle.

For each polymer, the surface water density was found to be constant at low weight fraction of CUP particles and when the weight fraction was high enough to cause inter-molecular counterion condensation the density of surface water drops due to the decreased effective charge density as shown in Table 3.

**Figure 13:**
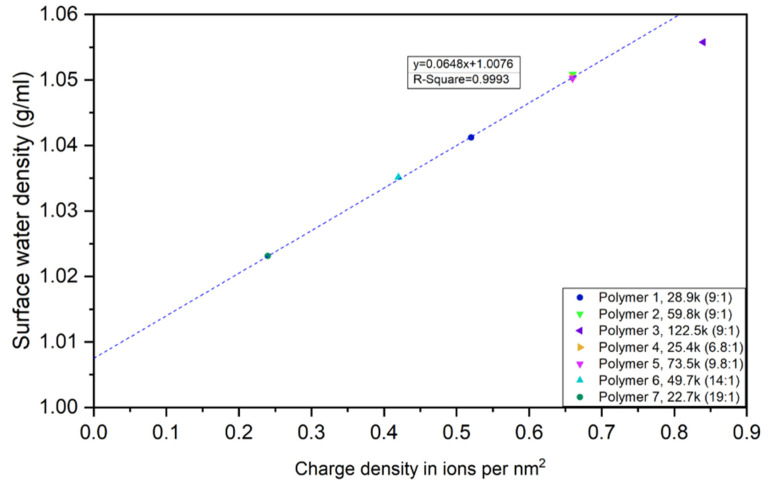
Surface water density vs charge density in ions per nm^2^.

When comparing the densities of surface water of CUP solutions with different molecular weights, as it is shown in Figure 13, Polymer 2, Polymer 4 and Polymer 5 have same charge density (0.66 charges per nm^2^) and the densities of surface water are the same. CUPs with higher charge density have higher surface water density. All the CUP polymers except Polymer 3 fit a linear regression relationship of surface water density to surface charge density. Polymer 3 has the largest molecular weight and highest charge density which results in intra-molecular counterion condensation reducing the surface effective charge. This condensation leads to the actual effective charge density being smaller, thus resulting in a slightly smaller surface water density than expected by the linear relationship. When extrapolating the linear function to zero, where there is no charge on the surface, the surface water density is 1.0076 g/mL, which is slightly larger than free water (0.997043 g/mL) [73]. This zero point should also represent the association with the ester groups which is the other group capable of hydrogen bonding interaction with water.

The density of the surface water can be related to the hydrogen bonding of water to the carboxylates and the ester groups on the surface. The carboxylate groups form up to six strong hydrogen bonds and the esters four weaker. Therefore, since the carboxylate groups form a stronger interaction the density would be higher than with the ester groups. As the charge density increases, the number of carboxylates increase as does the density [74].

### 4.5. Surface Water Thickness

To gain a deeper understanding of CUP surface water, it is important to relate our results to a structural model for the calculation of the surface water layer thickness. The total surface area of the CUP particles was calculated based on the measurement of the diameter of the CUP particle. The densities of surface water were determined, by knowing the weight fraction of surface water and number of CUP particles, the thickness of each sample was determined by Equation (12) [8].

(12) $$\frac{4}{3}\pi (\lambda +\frac{d}{2}{)}^{3}-\frac{4}{3}\pi {\left(\frac{d}{2}\right)}^{3}=\frac{X_{SW}M}{X_{CUP}N_{A}\rho_{SW}},$$

where λ is the thickness of surface water, d is the diameter of CUP particle, X_SW_ is the weight fraction of surface water, X_CUP_ is the weight fraction of CUP particle, M is the molecular weight of CUP, N_A_ is Avogadro constant, ρ_sw_ is the density of surface water.

Table 4 shows the surface water thickness of each CUP at different weight fractions. The thickness of CUPs ranges from 0.43 to 0.77 nm, which is about two to four water molecules thick. While previous studies have been suggested that surface water is as thick as from a few water molecules to several dozens of water molecules depending on the particles surface properties [75,76]. The thickness is likely to be primarily controlled by the charge density of the CUP particle surface and any hydrogen bonding groups at the surface. Charges will hold the water in a more bound state than water more loosely held at more hydrophobic surface groups. Comparing Polymer 1, 2 and 3, it can be concluded that the larger particle has the thicker surface water layer due to it having more carboxylate groups at the surface per unit area. A higher charged surface will form a thicker electrical double layer, giving more counterions and associated more water molecules. This result agrees with the findings of Chen et al. [66]. While Polymer 4 has a lower molecular weight but the same ions per nm^2^ as Polymer 2, the thickness of surface water are the same. By looking at Polymer 1, the thickness of surface water is 0.636 nm when the weight fraction is small and the thickness decreases to 0.564 nm when the weight fraction is 25% due to the increased inter-molecular counter-ion condensation. This again implies that, the thickness of surface water is proportional to ions per nm^2^, which also corresponds to the finding of weight fraction of surface water.

**Figure 14:**
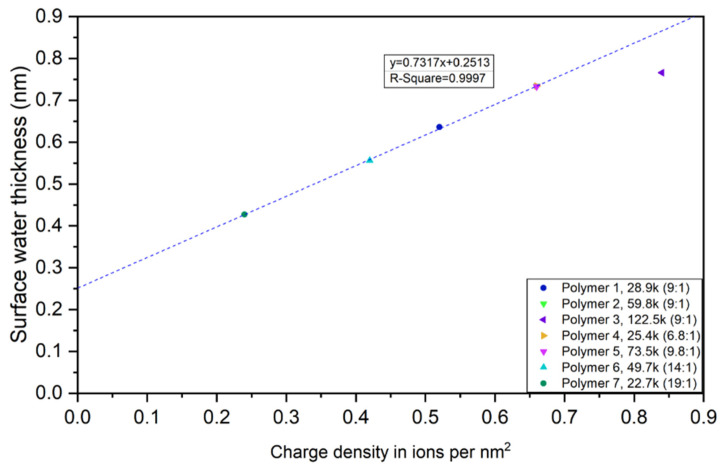
Surface water thickness vs charge density in ions per nm^2^.

Figure 14 shows the plot of surface water thickness and charge density in ions per nm^2^. When the charge density increases on a single CUP particle, the intra-molecular counterion condensation occurs on itself, reducing the effective charges on the surface, resulting in a thinner surface water layer. When charge density was smaller than 0.66 nm, surface water thickness increases with the increasing of charge density and when charge density is higher, surface water thickness increases but less than expected due to the inter-molecular counterion condensation. Extrapolation of the line to zero shows that when there is no effective charge on CUP surface, there is still a 0.2513 nm surface water layer, which is approximately a monolayer of water [77,78,79].

### 4.6. Melting Point Depression

The melting point depression of CUP solutions can also offer potential information. DSC was used to accurately measure the heat flow associated with thermal transitions in CUP solutions. As is well known, by adding a non-volatile solute, the melting point of a solvent decreases [80]. If we consider the solution is ideal, the freezing/melting point depression can be described by Equation (13).

(13) $$\Delta T_{F}=K_{F}\cdot b\cdot i,$$

where ΔT_F_ is the melting point depression in K, K_F_ is cryoscopic constant (1.853 K·kg/mol), i is van’t Hoff constant, b is the molality of solute in mol/kg.

Since the CUP solutions were modified by NaOH to pH = 8.5, it is important to know if the Na^+^ might contribute to the temperature depression, which might affect the van’t Hoff factor in Equation (13). The molality of Na^+^ in CUP solutions was calculated by Equation (14).

(14) $$b_{Na}=\frac{10^{pH-14}\cdot V}{m_{solution}-m_{NaOH}},$$

where b_Na_ is the molality of sodium ions, V is the volume of solution, msolution is the mass of solution, m_NaOH_ is the mass of NaOH.

The measured melting point depression of water and NaOH modified water was obtained by DSC, there was no difference between pure and pH modified water. By knowing the molality of Na^+^, the melting point depression, contributed by sodium ion in solution, was calculated to be 1.1755×10^−5^ K, obtained from Equation (14), which was negligible.

Knowing the molality b=(n_solute_/m_solvent_), the molality of CUP particles was calculated from Equation (15).

(15) $$b_{CUP}=\frac{X_{CUP}}{(1-X_{CUP})\cdot M_{W}},$$

where b_CUP_ is the molality of CUP particle, M_W_ is the molecular weight of CUP, X_CUP_ is the weight fraction of CUP.

**Figure 15:**
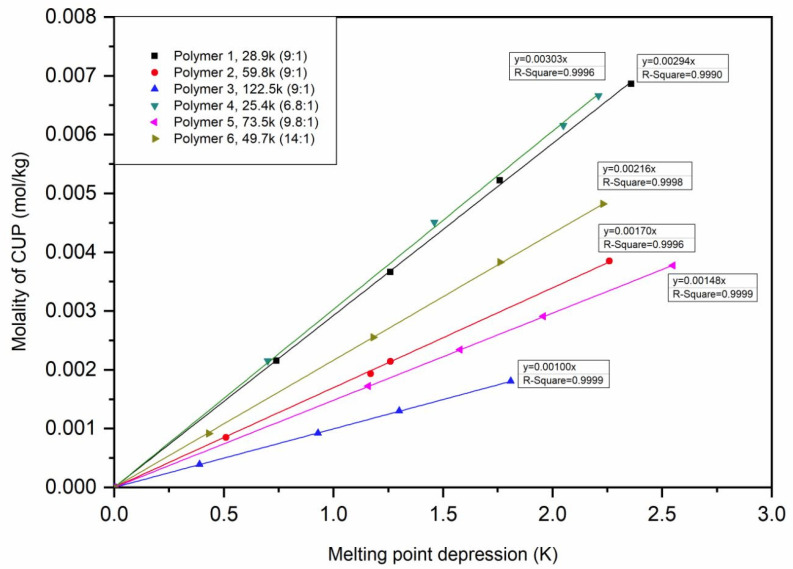
Molality of CUPs vs melting point depression.

By using Equation (15), the molality of CUP can be calculated, the relation between the molality of CUP and melting point depression was plotted in Figure 15.

The molality of CUP being equal to 0.001 mol/kg was picked, the corresponding ΔT_CUP_ was determined from Figure 15. Rearranging Equation (13), the van’t Hoff factor can be described as Equation (16).

(16) $$i=\frac{\Delta T_{CUP}}{b\cdot K_{F}}.$$

In each CUP particle, the number of repeat units (n_rep_) was calculated by dividing the molecular weight of CUP by the molecular weight of each repeat unit, Equation (17).

(17) $$n_{rep}=\frac{M_{W}}{n\cdot M_{MMA}+M_{MAA}},$$

where n_rep_ is the number of repeat unit in one CUP particle, M_MMA_ is the molecular weight of MMA monomer, M_MAA_ is the molecular weight of MAA monomer, n is the molar ratio of monomer MMA/MAA.

The number of effective groups that contributed to van’t Hoff factor can be expressed as Equation (18).

(18) $$n_{eff}=\frac{i}{n_{rep}},$$

where n_eff_ is the number of effective groups in each repeat unit.

The van’t Hoff factor, which is the number of ions per individual molecules of solute. As it was well known, van’t Hoff factor is equal to 2 for NaCl and 3 for BaCl_2_ as an example. In each repeat unit, there is one carboxylate group and several ester groups. Each carboxylate group was neutralized by NaOH, thus sodium ion may or may not be taken into account, shown in Equations (19) and (20).

(19) $$n_{ester}=n_{eff}-n_{c}$$

(20) $$n_{ester}=n_{eff}-2\cdot n_{c},$$

where n_c_ is the number of carboxylate group in each repeat unit, which equals to 1, n_ester_ is the number of ester group in each repeat unit.

The surface area of the CUP particle can be expressed as A_CUP_ = 4πr^2^, average area of each repeat unit on CUP was determined.

(21) $$A_{rep}=\frac{A_{CUP}}{n_{rep}}.$$

Since the surface area of CUP was taken by carboxylate and ester groups, in each repeat unit, there is one carboxylate group and several ester groups. Using Equation (22), the average area of each ester group and carboxylate group were determined by using different molecular weight CUP particles.

(22) $$A_{rep}=A_{c}+n_{ester}\cdot A_{ester},$$

where A_c_ is the average area of one carboxylate group occupied on CUP surface, A_ester_ is the average area of one ester group occupied on CUP surface.

The calculation indicated that sodium ion did contribute to the van’t Hoff factor value, the average area of an ester group on CUP surface was 0.374 nm^2^, while average area of a carboxylate group is 0.287 nm^2^. If assuming each group is a plane circle, the radius of carboxylate group is 0.244 nm, the radius of carboxylic acid group is 0.213 nm. Which is similar to the total of the length of C=O and O–H bond. The number of carboxylate groups varies upon different molecular weight and charge density of CUP particles. Results are shown in Table 5 [81,82].

Polymer 1, 2 and 3 have the same monomer ratio, with the increasing of molecular weight of CUP, the number of ester groups per repeat unit decreases. Because higher molecular weight CUP has more of hydrophobic groups on the inside, leaving more hydrophilic charged groups on the surface, giving higher ions per nm^2^. Polymer 2, 4 and 5 have similar ions per nm^2^ but different molecular weight, the number of ester groups per repeat unit are fairly close. This again indicates that the number of ester groups on the surface is dependent on the charge density with the carboxylates taking surface positions over esters during the formation of CUPs. The implication is that the area of the surface minus the area needed by the carboxylates leaves the remainder to be filled in by the esters with most esters being on the particle interior.

### 4.7. Specific Heat Analysis

Specific heat is also an important thermodynamic component. The specific heat measurement at a given temperature were also gathered from the DSC measurement, however, in order to obtain proper and critical measurement, the zeroline has to be measured separately and subtracted from the measured curve before evaluation [83]. An empty DSC pan was used to determine the heat flow rate of the zeroline ɸ_0_ (T) and a calibration substance of a known behavior, water, was placed into another DSC pan with similar mass, using the same experimental procedure. The precise specific heat C_S_ can be calculated by simply subtracting the calibrated heat flow rate zeroline and combing with the known substance, shown in Equation (23).

(23) $$C_{S}=\frac{q_{S}-q_{0}}{q_{ref}-q_{0}}\cdot \frac{m_{ref}}{m_{S}}\cdot C_{ref},$$

where C_S_ is the specific heat of sample, C_ref_ is the specific heat of reference, q_s_ is the heat flow rate of the sample, q_0_ is the heat flow rate of empty DSC pan, q_ref_ is the heat flow rate of reference, m_ref_ is the mass of reference, m_s_ is the mass of sample.

If considering the mass difference of two DSC pans used for zeroline calibration and actual measurement, it is possible to further make routine calculation, Equation (24). However, the DSC pan correction result in an error smaller than 1%, which is negligible.

(24) $$C_{S}=\frac{q_{S}-q_{0}}{q_{ref}-q_{0}}\cdot \frac{m_{ref}}{m_{S}}C_{ref}+\frac{m_{Cr,ref}-m_{Cr,S}}{m_{S}}\cdot C_{Cr},$$

where m_Cr, ref_ is the mass of reference DSC pan, m_Cr, S_ is the mass of sample DSC pan.

The dry polymer is a fine powder with low thermal conductivity, in order to have better thermal conductivity, the dry polymer was put in an open cap DSC sample pan and heated to 453.15 K to get rid of the moisture and let the polymer melt to have better contact with the pan. The pan was then sealed, cooled to 233.15 K, isothermal for 10 min, heated to 313.15 K. Two temperatures were picked for measurement, 253.15 K and 293.15 K, because at 253.15 K, the free water is in its ice form, while at 293.15 K, free water is liquid. Also, at these two temperatures, the baseline is strait, there is no overlapping with the endothermic peak and for one sample at a fixed temperature, the specific heat of dry CUP and surface water are constant. The specific heat of CUP solution is the total of the specific heat of free water (ice), dry CUP and surface water. The specific heat of each component at 263.15 K and 293.15 K was obtained from DSC measurement. Knowing the weight fraction, the specific heat of surface water was determined by Equations (25) and (26).

(25) $$C_{P(s)}=aC_{P(ice)}+bC_{P(CUP)}+cC_{P(sw)}$$

(26) $$C_{P(s)}=aC_{P(Fw)}+bC_{P(CUP)}+cC_{P(sw)},$$

where C_P(s)_ is the specific heat of CUP solution, C_P(ice)_ is the specific heat of ice, C_P(CUP)_ is the specific heat of CUP particle, C_P(sw)_ is the specific heat of surface water, C_P(Fw)_ is the specific heat of free water, a, b and c represent the weight fraction of each component in the CUP solution.

The specific heat of each component in Polymer 1–7 were calculated, surface water specific heat is about 3.07 to 3.09 J/g·K at 293.15 K and 3.04 to 3.07 J/g·K at 253.15 K, which exhibit a small dependency on charge density, Table 6. The values exhibit only a small decrease in going from liquid water temperatures to well below freezing for water. The specific heat of surface water was independent of concentration for the range studied, which were all below the point of Manning condensation. The data for surface water at 293.15 K indicates that the water has more freedom than ice but less than liquid water. The small lowering of the specific heat with a 40 degree drop was similar to many materials not undergoing a phase change or other transition in mobility.

Surface water associated to the carboxylate and ester groups on CUP surface, known the specific heat of surface water at 293.15 K and 253.15 K, as well as the number of each functional groups, the specific heat of water associated to the carboxylate and ester groups could be estimated by Equation (27).

(27) $$C_{P(sw)}=\frac{n_{c}}{n_{c}+n_{ester}}\cdot C_{P(swc)}+\frac{n_{ester}}{n_{c}+n_{e}{}_{setr}}\cdot C_{P(swe)},$$

where C_P(swc)_ is the specific heat of water associated to carboxylate groups, C_P(swe)_ is the specific heat of water associated to ester groups.

Table 7 shows the specific heat due to the ester and the carboxylate groups on the surface. This estimation of the effect of each group gives a relatively consistent average value which could be used to define a new polymer based on a group contribution basis.

## 5. Conclusions

This work discussed the thermal properties of CUPs with different molecular weight, monomer ratio and CUP surface charge density (ions per nm^2^), based on the heat of fusion, specific heat and melting point depression aspects. It was found that surface water occupied a significant amount of volume in CUP solutions. Rapid cooling of CUP solutions will result in larger amount of surface water due to more rapid ice crystal growth and less time for CUP particles to migrate and undergo Manning condensation. The effect of cooling rate is less on the higher weight fraction solutions due to charge–charge repulsion which lowers mobility. The density of surface water was calculated and ranged from 1.023 g/mL to 1.056 g/mL depending on the charge density. The thickness of surface water was calculated, showing a trend of increasing with the increasing surface charge density. The molecular weight had no effect on the thickness of the surface water layer. However, as the weight fraction of CUP particles increased above approximately 20%, inter-molecular counterion condensation occurs and decreases surface water layer thickness. The melting point depression was found to linearly dependent upon molality of CUPs with the slope being related to the number of ions on the surface of CUPs. Using the melting point depression data, the average area of carboxylate and ester groups were determined and its results are independent of the molecular weight. The specific heat of surface water was found to be 3.07 to 3.09 J/g·K at 293.15 K and 3.04 to 3.07 J/g·K at 253.15 K, which was between ice and free water and exhibited a small dependency with the surface charge density. CUPs are true nanoscale spheroidal particles with the molecular weight and surface charge being easily designed and synthesized. The findings can be readily extended and utilized in many fields including biochemical and life sciences. Therefore, CUP particles offer an excellent model to investigate the behavior of surface water, which can be of fundamental importance to protein, micelle, hydrogel and material science. In addition, CUP solution is zero VOC and free of surfactant, it has great potentials in coating, adhesive and many other applications. This work represents a comprehensive study of CUP surface water which defines structural components and their effects on surface water in a detailed quantitative manner for the first time.

## Figures and Tables

**Table 1:** Polymer synthesis: the amount of materials used.

Polymer	Molecular Weight (g/mol)	Monomer 1	Monomer 2	Initiator	Chain Transfer Agent	Solvent
		MMA (mol)	MAA (mol)	AIBN (mol)	1-Dodecanethiol (mol)	THF (mol)
1	28,900	2.25	0.25	1.75×10^−3^	7.61×10^−3^	10.40
2	59,800	2.25	0.25	1.75×10^−3^	3.79×10^−3^	10.40
3	122,500	2.25	0.25	1.75×10^−3^	1.87×10^−3^	10.40
4	25,400	2.22	0.33	1.78×10^−3^	8.62×10^−3^	10.40
5	73,500	2.30	0.23	1.77×10^−3^	3.10×10^−3^	10.40
6	49,700	2.35	0.17	1.76×10^−3^	4.51×10^−3^	10.40
7	22,700	2.39	0.13	1.76×10^−3^	9.46×10^−3^	10.40

**Table 2:** Molecular weight, particle size, acid number and density of the polymers.

Sample ID	M_n_ (g/mol)	Monomer Ratio	Particle Size (nm)	AN (mg KOH/g)	Density of Dry CUP, ρ_p_ (g/mL)	Charge Density in Ions per nm^2^
Polymer 1	28,900	9:1	4.22	56.8	1.2246 ± 0.0018	0.52
Polymer 2	59,800	9:1	5.38	57.0	1.2311 ± 0.0014	0.66
Polymer 3	122,500	9:1	6.83	56.9	1.2342 ± 0.0018	0.84
Polymer 4	25,400	6.8:1	4.04	73.2	1.2243 ± 0.0018	0.66
Polymer 5	73,500	9.8:1	5.76	52.6	1.2315 ± 0.0018	0.66
Polymer 6	49,700	14:1	5.06	37.7	1.2307 ± 0.0016	0.42
Polymer 7	22,700	19:1	3.90	28.2	1.2241 ± 0.0018	0.24

**Table 3:** Density of surface water for Polymers 1–7.

CUPs	X_CUP_	ρ_sw_ (g/mL)	CUPs	X_CUP_	ρ_sw_ (g/mL)	CUPs	X_CUP_	ρ_sw_ (g/mL)
1 28.9k (0.52)	5.85%	1.0413	3 122.5k (0.84)	4.53%	1.0544	5 73.5k (0.66)	11.19%	1.0491
	9.57%	1.0411		10.12%	1.0560		14.67%	1.0502
	13.32%	1.0412		13.72%	1.0564		17.62%	1.0506
	16.55%	1.0412		18.10%	1.0563		21.72%	1.0512
	25.01%	1.0358						
2 59.8k (0.66)	4.83%	1.0511	4 25.4k (0.66)	5.18%	1.0500	6 49.7k (0.42)	4.35%	1.0335
	10.35%	1.0508		10.15%	1.0500		11.25%	1.0354
	11.35%	1.0509		13.41%	1.0502		16.11%	1.0357
	18.72%	1.0507		14.30%	1.0501		19.32%	1.0360
7 22.7k (0.24)	5.00%	1.0231						

**Table 4:** Surface water thickness of each CUP at different concentrations.

CUPs	X_CUP_	λ (nm)	CUPs	X_CUP_	λ (nm)	CUPs	X_CUP_	λ (nm)
1 28.9k (0.52)	5.85%	0.635	3 122.5k (0.84)	4.53%	0.766	5 73.5k (0.66)	11.19%	0.734
	9.57%	0.637		10.12%	0.766		14.67%	0.733
	13.32%	0.636		13.72%	0.765		17.62%	0.734
	16.55%	0.636		18.10%	0.766		21.71%	0.732
	25.01%	0.564						
2 59.8k (0.66)	4.83%	0.733	4 25.4k (0.66)	5.18%	0.732	6 49.7k (0.42)	4.35%	0.556
	10.35%	0.735		10.15%	0.735		11.25%	0.556
	11.35%	0.734		13.41%	0.734		16.11%	0.557
	18.72%	0.735		14.30%	0.735		19.32%	0.556
7 22.7k (0.24)	5.00%	0.427						

**Table 5:** Average area of each carboxylate and ester group on CUPs.

CUPs	ΔT_CUP_ (K)	b_cup_	i	n_rep_	n_eff_	A_rep_ (nm^2^)	n_ester_	Ae (nm^2^)	Ac (nm^2^)	n*_c_/n*_ester_ per CUP
1 (0.52)	0.344	0.001	185.645	29.276	6.341	1.911	4.341	0.375	0.284	29.3/126.3
2 (0.66)	0.589	0.001	317.863	60.579	5.247	1.501	3.247	0.374	0.285	60.6/196.7
3 (0.84)	1.010	0.001	545.062	124.096	4.392	1.181	2.392	0.375	0.285	124.1/296.8
4 (0.66)	0.330	0.001	178.090	33.121	5.377	1.548	3.377	0.373	0.289	33.1/111.9
5 (0.66)	0.674	0.001	363.734	68.869	5.282	1.513	3.282	0.374	0.286	68.9/226.0
6 (0.42)	0.462	0.001	249.325	33.406	7.463	2.408	5.463	0.372	0.290	33.4/182.5
7 (0.24)	0.262	0.001	141.392	11.417	12.397	4.185	10.397	0.375	0.290	11.4/118.7

ΔT_CUP_: melting point depression in K, determined from Figure 15. b_CUP_: molality of CUP particles. i: van’t Hoff factor. n_rep_: number of repeat unit in each CUP particle. n_eff_: number of effective groups per repeat unit. n_ester_: number of ester groups in each repeat unit. A_rep_: area of one repeat unit. A_e_: average area each ester group takes (in equation ΔT= i·K_F_·b, consider i = 2). A_c_: average area each carboxylate group takes (in equation ΔT= i·K_F_·b, consider i= 2). n*_c_/n*_ester_: number of carboxylate and ester groups on one CUP particle surface.

**Table 6:** Specific heat of each components in polymer 1–7 (J/g·K).

CUPs	X_CUP_	CUP Solid (253.15 K)	Surface Water (253.15 K)	Ice (253.15 K)	CUP Solid (293.15 K)	Surface Water (293.15 K)	Free water (293.15 K)
1 28.9k (0.52)	5.85%	1.235	3.055	1.943	1.321	3.081	4.182
	9.57%	1.235	3.052	1.943	1.321	3.078	4.182
	13.32%	1.235	3.053	1.943	1.321	3.082	4.182
	16.55%	1.235	3.053	1.943	1.321	3.080	4.182
2 59.8k (0.66)	4.83%	1.230	3.047	1.943	1.315	3.073	4.182
	10.35%	1.230	3.051	1.943	1.315	3.076	4.182
	11.35%	1.230	3.049	1.943	1.315	3.072	4.182
	18.72%	1.230	3.049	1.943	1.315	3.074	4.182
3 122.5k (0.84)	4.53%	1.195	3.042	1.943	1.305	3.067	4.182
	10.12%	1.195	3.041	1.943	1.305	3.068	4.182
	13.72%	1.195	3.040	1.943	1.305	3.065	4.182
	18.10%	1.195	3.041	1.943	1.305	3.066	4.182
4 25.4k (0.66)	5.18%	1.235	3.050	1.943	1.322	3.073	4.182
	10.27%	1.235	3.049	1.943	1.322	3.075	4.182
	13.52%	1.235	3.050	1.943	1.322	3.074	4.182
	14.47%	1.235	3.051	1.943	1.322	3.076	4.182
5 73.5k (0.66)	11.23%	1.228	3.048	1.943	1.311	3.074	4.182
	14.67%	1.228	3.047	1.943	1.311	3.075	4.182
	17.62%	1.228	3.049	1.943	1.311	3.074	4.182
	21.71%	1.228	3.048	1.943	1.311	3.075	4.182
6 49.7k (0.42)	4.35%	1.231	3.059	1.943	1.316	3.086	4.182
	11.25%	1.231	3.058	1.943	1.316	3.087	4.182
	16.11%	1.231	3.059	1.943	1.316	3.086	4.182
	19.32%	1.231	3.061	1.943	1.316	3.086	4.182
7 22.7k (0.24)	5.00%	1.237	3.066	1.943	1.324	3.093	4.182

**Table 7:** Specific heat of surface water associated with carboxylate and ester groups (J/g·K).

CUPs	Cp_(SW)_ of Carboxylate (293.15 K)	Cp_(SW)_ of Ester (293.15 K)	Cp_(SW)_ of Carboxylate (253.15 K)	Cp_(SW)_ of Ester (253.15 K)
1 (0.52)	2.979	3.103	2.965	3.073
2 (0.66)	2.979	3.103	2.954	3.078
3 (0.84)	2.984	3.102	2.953	3.078
4 (0.66)	2.965	3.107	2.956	3.078
5 (0.66)	2.975	3.105	2.965	3.073
6 (0.42)	2.960	3.109	2.954	3.078
7 (0.24)	2.978	3.104	2.956	3.077
